# Progress in Surgery

**Published:** 1898-04-23

**Authors:** 


### Progress in Surgery.

### SURGERY OF THE THYROID GLAND.

**Exophthalmic Goitre.**—The surgical treatment of exophthalmic goitre formed one of the subjects for discussion at the Surgical Congress in Paris, thyroidectomy, ligature of the thyroid arteries, and operations on the cervical sympathetic being separately considered. Jonnesco showed that the mortality is nearly 20 per cent. when thyroidectomy is performed, whilst the complete cures are less than 40 per cent. However, the mortality in typical cases of Graves's disease is very much higher than that given, and the rapidity and unavoidability of death are lamentable. Doyen expressed himself as somewhat favourable to thyroidectomy.

As regards ligature of the thyroid arteries, Kocher had operated thirty-four times, with a mortality of about 9 per cent., and the rest were either cured or relieved.

Ligature of the cervical sympathetic had been done three times by Faure. One case improved, another nearly died on the table from syncope, and the third actually did die from heart failure during the operation.[1] A successful case was reported by Gérard-Marchant and Abadie. The patient appeared to be well at the end of ten days; this contrasts with another case as markedly unsuccessful.[2] The results do not altogether confirm the favourable opinion held by Jaboulay, who reported nine successful cases, and stated that the operation had never been followed by ill effects of any kind, there having been no trophic disturbances, no changes in any organ or tissue, and no impairment in vesical accommodation. He thinks that the results are better in the aged.

A fatal case of thyroidectomy for Graves's disease was recorded by Paul.[3] The operation was perfectly straightforward. The patient became restless, and pulse rapid, and she died two and a half days after the operation. Another case nearly ended fatally, but appeared to improve after some pent-up, thin watery fluid was removed from the wound. Paul attributes these untoward results to grasping and manipulating the gland, and he states that his results were better when he ligatured the isthmus, and took care to avoid excessive manipulation.

The observations of Schulz seem to show that partial thyroidectomy, even with severe cases, is attended with much less serious consequences, and little tendency to recurrence. Cases have been watched for two to seven years. A case in contrast has been described by Lejars, where a young woman died within a few hours of operation.

**Sarcoma of the Thyroid.**—Two cases have been placed on record of primary sarcoma of the thyroid gland. In one case the tumour was irremovable, so the isthmus was incised and the contents of each lateral lobe were curetted out.[4] The other case is of interest from the fact that, although the operation of removal of the entire gland was performed under cocaine, without a general anæsthetic, yet the patient felt very little pain and conversed with the surgeon during the operation.[5] The patent became collapsed, and died six hours after the operation. The author suggests the desirability of using cocaine rather than chloroform when the breathing is likely to be embarrassed by the pressure of the growth.

[1] Birm. Med. Review, Mar., 1898. [2] Amer. Jour. Med. Science, Jan, 1898. [3] Brit. Med. Jour., Jan. 1, 1898. [4] Annals of Surg., Oct, 1897. [5] Annals of Surg., June, 1897.

### NEW GROWTHS.

**Special Tumours.**—Mr. Bland Sutton[1] has recently described some cases of tumour of more than ordinary interest. The first case was one of chondrana of the submaxillary gland. When first observed it was the size of a cherry; when removed at the end of forty-four years it had become so large that the patient lodged it upon her shoulder for relief from its weight. It was successfully removed. The writer refers to the fact that cartilage is fifty times more frequent in the parotid than in the submaxillary gland, and it is doubtful if it ever occurs in the sublingual gland or in the pancreas. It has been observed in the lachrymal gland rarely.

Another case was one in which a pedunculated and much lobulated tumour hung from the nipple of a woman aged forty. It was congenital in origin, and microscopically had the character of the tumours in molluscum fibrosum whether of the pedunculated type, or of the large redundant fold type, or of the multiple discrete nodular type. A third case is described in which a large fibro-fatty tumour grew for thirty years in the plantar fascia of the foot and led to amputation. Such a situation for a fatty tumour is distinctly rare. Fourthly, dermoids arising in the median line of the nose are described. Those below the bridge are always in the mid line, and are formed by inclusion during the fusion of the rounded inferior angles of the fractc-nasal plate, the globular processes. Each of these processes forms a portion of the ala of a nostril and the corresponding premaxilla. Those on the bridge of the nose are formed in the same manner as those which occur on the scalp.

**Fibro-sarcoma of the Sciatic Nerve.**—Mr. James Berry has put on record an interesting case of the above nature. The patient, a woman nineteen years of age, had a swelling at the back of the left thigh extending from just above the knee to the level of the trochanter, and measuring twelve inches vertically. There was very little pain from the tumour, unimpaired mobility of the hip and knee joints, and no wasting of the leg muscles. There were no glands. It was dissected out through an incision about sixteen inches in length. Four inches of sciatic nerve were much inflated, and the upper parts of the hamstrings. The latter were entirely removed and the affected portion of the sciatic. This patient recovered, and was able to walk without the aid of crutch or stick. Sensation returned to the foot, and foot-drop was prevented by the use of a gutta-percha splint. The growth was in part fibrous, in part sarcomatous. It is remarkable that so little pain is produced in this class of tumour. The patient retained power of knee flexion which was affected by the sartorius.

**Lipomata.**—Dr. Meyer and Dr. Weir[2] each have recently operated for those rare forms of lipoma which arise deeply in close relation to the periosteum of bones. In Meyer's case the growth probably originated between the anterior surface of the femur and the supra-patella pouch of the knee-joint capsule. That joint had to be opened for the removal of the growth. Full power over the limb was regained. Weir's case lay over the fibula, and was largely contained in the extensor lingus digitorum muscle. It weighed two pounds, and was successfully removed. Both these cases closely similated sarcomata in their clinical aspect.

**Cysts and Cystic Tumours of the Breasts.**—Sasse,[3] after a careful pathological and clinical study of a series of cases, classifies cystic tumours of the breast into two principal divisions: (1) Those produced by a chronic interstitial inflammation, and resulting in a widening of the excretory ducts into cysts—mastitis chronica cystica. (2) Multiple cysts resulting from a pure epithelial hypertrophy and cystic dilatation of the acini-polycystoma mammæ. They have the same origin as the small cysts which are found in the sound portion of a breast hardened by carcinomatous disease in profuse numbers. These cysts, on the other hand, can easily change into carcinoma. General cystic disease of the mamma, described originally by Brodie,[4] is now generally considered to be a late stage of chronic interstitial mastitis, common in women nearing the climacteric. Another form of general cystic disease has been alluded to by Mr. Marmaduke Shield.[5] It is characterised by the development of a number of papilomatous growths within the dilated ducts and tubules, and blood-stained discharge from the nipple usually follows. The so-called serous cyst of the mamma is by some regarded as formed in lymphatic spaces, by others as formed in acini, and comparable to renal cysts.

**Suppurating Chondro-Osteosarcoma of Breast.**—W. Arnold, of Lucerne, describes a case of this kind.[6] The patient was 67. The swelling was first diagnosed as an abscess, and incised, pus escaping. The swelling, however, increased, with fever, and extension of inflammation to the circumference of the breast. In the midst of the swelling was a thick walled cavity which discharged pus. Glands were not affected. Recovery followed amputation. Examination showed the tumour to be a sarcoma, containing bone and cartilage.

[1] Practitioner, Nov., 1897. [2] Annals of Surgery, Aug. [3] American Journ. Med. Science, Sept. [4] Practitioner, July. [5] Clinical Journ., Dec., 1897. [6] B. Med. Jour., Vol. II., 1897.

